# MicroRNA-17 inhibits tumor growth by stimulating T-cell mediated host immune response

**DOI:** 10.18632/oncoscience.69

**Published:** 2014-07-27

**Authors:** Haoran Li, Shaan Gupta, William W. Du, Burton B. Yang

**Affiliations:** ^1^ Sunnybrook Research Institute, Sunnybrook Health Sciences Centre, Toronto; ^2^ Department of Laboratory Medicine and Pathobiology, University of Toronto, Toronto

**Keywords:** microRNA, miR-17, T-cell, Stat3, immune response

## Abstract

**Background:**

Melanoma is one of the fastest-rising types of cancer in North American. Accumulating evidence suggests that anti-tumor immune tolerance plays a critical role in tumor development.

**Methods:**

B16 melanoma cells were injected into wild type and miR-17 overexpressing transgenic mice. Tumor growth was monitored and tumor bearing mice were sacrificed by the end of the forth week. Peripheral blood and spleen cells were subject to flow cytometry analysis and tumor samples were subject to immunohistochemistry staining. Meanwhile, Jurkat cells transfected with mock-control or miR-17 overexpressing plasmid were co-cultured with B16 cells. The influence of miR-17 on cell cycle, proliferation and survival was evaluated.

**Results:**

The melanoma tumors formed in mice overexpressing miR-17 were less than that in wild type mice. In addition, the miR-17 tumors were less invasive and less angiogenic. The percentage of CD8+ T cells was suppressed in miR-17 transgenic mice before melanoma cell injection. Its level was significantly increased upon tumor grafting. More tumor infiltrating CD8+ cytotoxic T lymphocyte could be found in transgenic mice with tumor formation. Luciferase assay and protein analysis indicated that STAT3 was the target of miR-17. Decreased levels of STAT3 were associated with miR-17 over-expression. Down-regulation of STAT3 in Jurkat cells promoted cell proliferation and mitosis.

**Conclusions:**

MiR-17 inhibits melanoma growth by stimulating CD8+ T cells mediated host immune response, which is due to its regulation of STAT3.

## INTRODUCTION

Melanoma is the most aggressive skin cancer, and is characterized by its rapid growth and early metastasis. It accounts for over 75% of deaths related to skin cancer. Melanoma has one of the fastest growing incidences in North America, and it has been steadily increasing for the past 30 years. It is estimated that 2% of Caucasians will develop melanoma in their lifetime [1]. In 2014, approximately 76,100 new cases will be diagnosed and about 9,710 individuals will die from melanoma in the United States [1]. Until 2011, there was no single agent available for the successful treatment of this disease. Owing to enormous progresses made in immunotherapy, many treatment options have emerged in recent years and the overall survival of patients with advanced melanoma has been significantly prolonged [2].

Immunotherapeutic drugs function by stimulating the host immune system, which recognizes and targets tumor cells in the tumor microenvironment. The tumor microenvironment has a pivotal role in the development and progression of tumors. The microenvironment comprises stromal cells, cytokines, signaling molecules and extracellular matrix. The interplay between the tumor and its surrounding microenvironment determines the balance between tumor growth and antitumor immune responses. Tumor cells are good at camouflage: they modify or shed their surface antigens to escape from immune surveillance. Therefore, overcoming immune tolerance will increase the effect of the antitumor immune response. By targeting molecules capable of manipulating the microenvironment, immunotherapy has emerged as a novel method to treat melanoma.

Melanoma cells harbor a multitude of gene mutations which favor tumor cell proliferation, invasion and metastasis. The signal transducer and activator of transcription (STAT3) protein is constitutively activated in approximately 50 to 90% of human cancers, including melanoma [3, 4]. Accumulating evidence suggests that elevated activity of STAT3 pathway is essential for the ability of melanoma cells to evade the immune system [5, 6]. STAT3 participates in tumor immune tolerance by inhibiting proinflammatory mediators and stimulating immune suppressing factors. As a result, T-cell functionality is suppressed and its immune response against tumor antigens is impaired. It is still poorly understood how T-cells, arisen from the human host, become tolerant to tumor cells. The restoration of infiltrative T-cell function in the tumor microenvironment may provide a potential therapeutic opportunity for overcoming the immune evasion of melanoma cells. Our studies show that STAT3 mediates the function of miR-17 in regulating T-cell activities, thus providing novel insight into the mechanisms that may underlie immune evasion in melanoma cells.

## RESULTS

### CD8+ cells increased in tumor-bearing miR-17 transgenic mice

**Figure 1:**
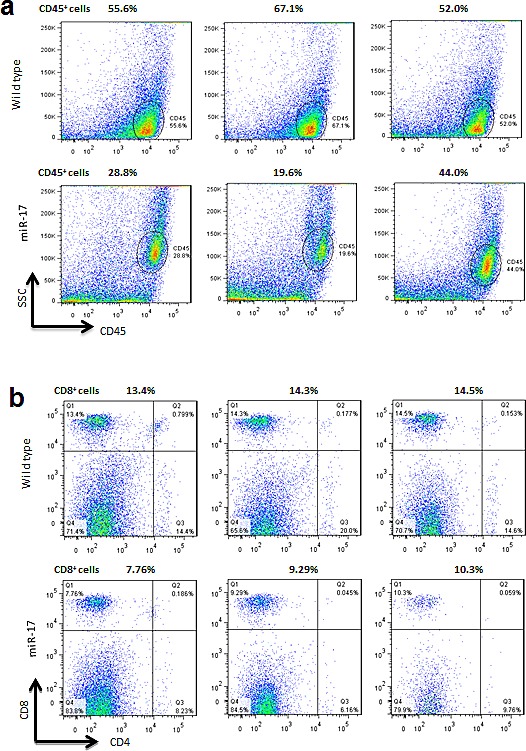
Analysis of cell subtype in non-tumor-bearing mice (A) Left, Lower amounts of CD45+ cells were detected in the miR-17 transgenic mice compared with the wild type mice (30.80% vs.58.23%, p=0.03). Right, Typical distributions of CD45 positive cells in the miR-17 transgenic and wildtype mice. (B) Left, The percentage of CD8+ cells was significantly less in transgenic mice than that in the wild type (9.12% vs. 14.07%, p<0.01). The percentage of CD4+ cells also decreased in miR-17 transgenic mice compared with wildtype (8.05% vs. 16.33%, p=0.02). The ratio of CD8+/CD4+ was decreased in transgenic mice (1.17 vs. 0.88, p=0.21). Right, Typical distributions of CD4, and CD8 subtypes in the miR-17 transgenic and wildtype mice.

Previous work from our lab showed that miR-17 is essential for hematogenesis and differentiation [7]. We therefore evaluated the influence of miR-17 on lymphopoiesis. CD45 is expressed on most hematolymphoid cells. We examined the number of CD45+ cells in peripheral blood and spleen. A lower percentage of CD45+ cells was detected in the miR-17 transgenic mice as compared with wildtype (30.80% vs. 58.23%, *p*=0.03) (Figure 1a). Since patients with melanoma containing a higher number of T lymphocytes show longer overall survival than those bearing tumors without T lymphocytes infiltrations [8], we analyzed the subpopulation of T lymphocytes including the number of CD8+ and CD4+ T cells in CD45+ cells (Figure 1b). C57BL/6 mice have a higher percentage of CD8+ cells, compared to other strains. The CD8/CD4 ratio was close to 1 in both wild type and miR-17 transgenic mice (1.17 vs. 0.88, *p*=0.21), which was consistent with previous findings [9]. However, the number of CD8+ cells was significantly less in the transgenic mice than that in wild type (9.12% vs. 14.07%, *p*<0.01). The number of CD4+ cells also decreased in the miR-17 transgenic mice compared to wildtype (8.05% vs. 16.33%, *p*=0.02).

**Figure 2:**
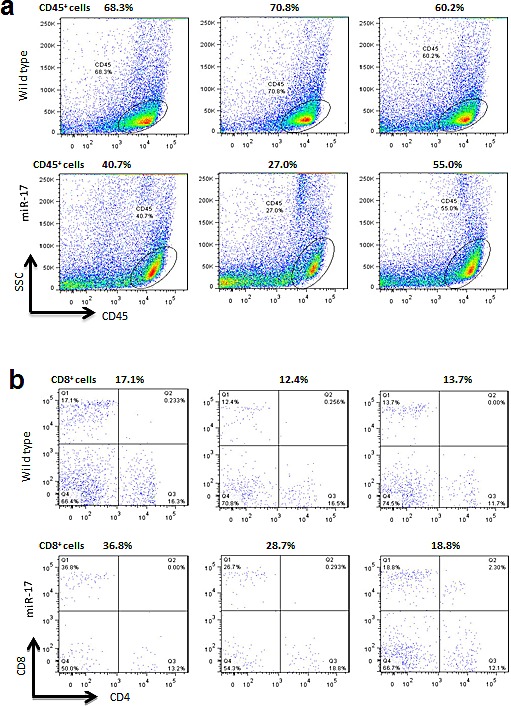
Cell subtype analysis in tumor-bearing mice (A) Left, Compared to the wild type mice, lower percentages of CD45+ cells were detected in the miR-17 transgenic mice (40.90% vs. 66.43%, p=0.04). Right, Typical distributions of CD45 positive cells in the miR-17 transgenic and wildtype mice. (B) Left, The percentages of CD8+ cells increased significantly in the miR-17 transgenic mice after tumor implantation compared with the non-tumor-bearing mice (27.43% vs. 9.12%, p=0.03). Nevertheless, there was little change in CD8+ cells in wild type mice (14.07% vs. 14.40% p=0.83). The ratio of CD8/ CD4+ cells in transgenic mice increased from 1.17 to 1.92 after tumor injection, while it only slightly increased from 0.88 to 0.99 in wild type controls. Right, Typical distributions of CD4, and CD8 subtypes in the miR-17 transgenic and wildtype mice.

We next injected mouse melanoma B16 cells intraperitoneally into wild type and miR-17 transgenic mice. On the 28th day, we collected blood in the periphery and the spleen from tumor-bearing mice. Compared to the mice without tumors, mice with grafted tumors had a higher number of CD45+ cells (Figure 1a and 2a). There was an 8% increase in tumor-bearing wild type mice compared to non-tumor mice (66.43% vs. 58.23%, *p*=0.21). In the miR-17 transgenic mice, an increase of 10% after melanoma implantation was detected (30.80% vs. 40.90%, *p*=0.40). In line with what we have seen in non-tumor-bearing mice, the population of CD45+ cells was relatively lower in tumor-bearing mice with miR-17 overexpression (40.90% vs. 66.43%, *p*=0.04) (Figure 2a).

Analysis of subpopulation of T cells showed that CD8+ cells were significantly increased in miR-17 transgenic mice after tumor implantation compared to non-tumor miR-17 mice (27.43% vs. 9.12%, *p*=0.03). Nevertheless, there was little change of CD8+ cells in wild type mice (14.07% vs. 14.40% *p*=0.83). The ratio of CD8/ CD4+ cells in transgenic mice increased from 1.17 to 1.92 after tumor injection, while it only slightly increased from 0.88 to 0.99 in wild type controls (Figure 2b). In summary, compared to the mice without tumor, a significant increase in CD8+ cells was observed in miR-17 overexpressing mice, but not in the wild type controls.

### Tumor invasion was inhibited in miR-17 transgenic mice

**Figure 3:**
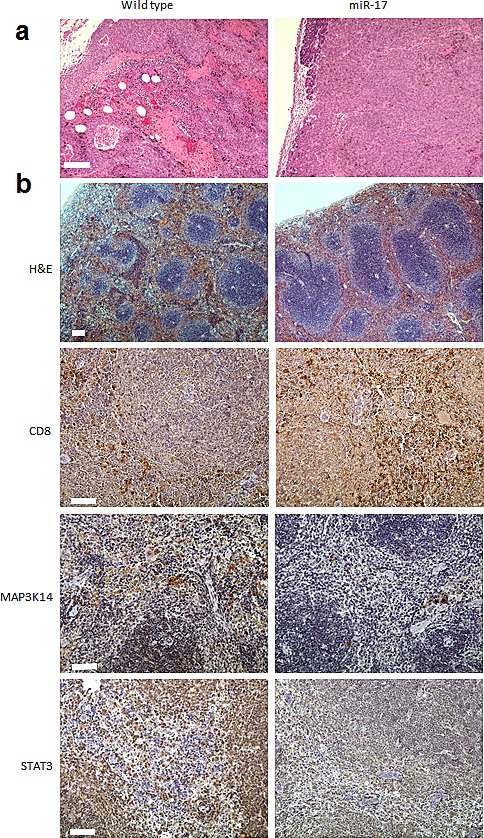
Immunohistochemistry analysis in B16 grafted tumor and host spleen (A) In the wild type mice, massive necrosis and internal bleeding could be found in the B16 melanoma, and tumor cells frequently invaded into stromal tissue. Meanwhile, intact plasma membrane and less hemorrhagic necrosis was seen in the miR-17 transgenic mice. Scale bar, 100 μm. (B) Enlarged white pulps could be seen in the transgenic spleen of tumor bearing mice (H&E staining). More CD8+ cells were in spleens of transgenic mice than that in the wildtype mice. Compared to the wild type mice, miR-17 overexpression in spleen was associated with reduced expression of MAP3K14 and STAT3. Scale bar, 100 μm.

When B16 cells were injected into the peritoneal cavities of miR-17 transgenic and wild type mice, they were capable of seeding on the surface of internal organs such as liver, bowels and omentum. In the wild type group, implantation metastasis was found in 84.6% of the mice, while only 40.0% of transgenic mice had seeded tumors (Chi square test, *p*=0.03). Tumor sections were stained with hematoxylin and eosin (H&E) after the mice were sacrificed by the end of fourth week. In the wild type mice, massive necrosis and internal bleeding could be found in the B16 melanoma, and tumor cells frequently invaded into stromal tissue (Figure 3a). In the miR-17 transgenic mice, grafted tumors were still surrounded by an intact plasma membrane and less hemorrhagic necrosis can be found inside tumor (Figure 3a). Accordingly, the sizes of tumors formed in the transgenic group were much smaller than that in the control group. Taken together, grafted melanoma cells were less invasive in the miR-17 transgenic mice than in the wild type mice.

We previously reported that decreased numbers and sizes of germinal centers were observed in non-tumor-bearing miR-17 transgenic mice [7]. We next examined the paraffin sections of spleen in tumor-bearing mice. By using H&E staining, enlarged white pulps could be seen in the transgenic spleen of tumor bearing mice (Figure 3b). Immunohistochemistry (IHC) analysis showed that more CD8+ cells were in the spleens of transgenic mice than in the wildtype mice (Figure 3b). We further examined expression of MAP3K14 and STAT3 in transgenic spleen. Compared to the wild type mice, miR-17 overexpression in spleen was associated with reduced expression of MAP3K14 and STAT3 (Figure 3b). Overall, provocative reactions were observed in the spleens of mice with miR-17 overexpression.

### MiR-17 targets STAT3 in melanoma tumor microenvironment

**Figure 4:**
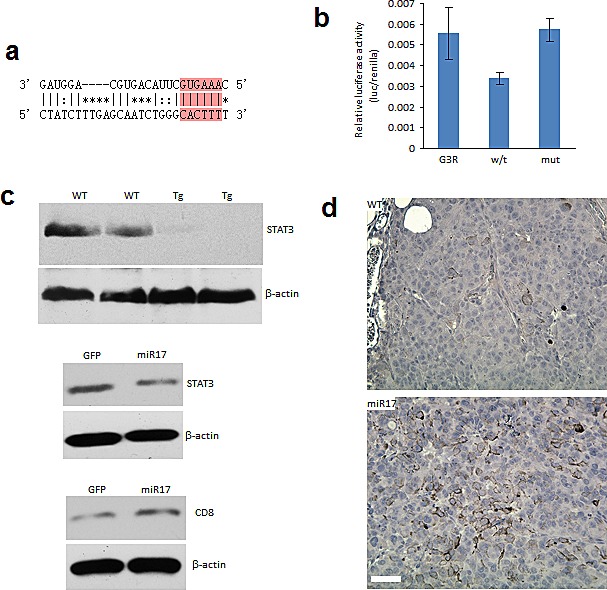
MiR-17 targets STAT3 in tumor stromal cells (A) A potential miR-17 target site was found in the 3′UTR of STAT3. (B) Luciferase assay confirmed that miR-17 was able to target the 3′UTR of STAT3. (C) Western blotting showed decreased expression of STAT3 in the miR-17 transgenic mice and in the miR-17-transfected Jurkat cells. CD8+ expression increased in the miR-17-overexpressed Jurkat cells. (D) Tumor infiltrating CD8+ cytotoxic T lymphocytes were increased in the miR-17-overexpressed transgenic mice. Scale bar, 50 μm.

Computational analysis showed that STAT3 is a candidate for miR-17 targeting. Its 3′-untranslated region (3′-UTR) contains a base pairing sequence complementary to the seed region of miR-17 (Figure 4a). We thereby designed a luciferase reporter construct which has a miR-17 binding site in the 3′-UTR of STAT3. In luciferase assay, miR-17 was able to bind to its complementary base pairing in luciferase reporter and reduced luciferase activity. We confirmed that mutation of the miR-17 binding site interfered with miR-17-target interaction, which led to restoration of luciferase activities (Figure 4b). As a result of miR-17 overexpression, the expression levels of STAT3 were suppressed in the spleens of transgenic mice (Figure 4c). Moreover, decreased expression of STAT3 was also detected in human T lymphocyte Jurkat cells transfected with the miR-17 overexpression plasmid (Figure 4c). Stable overexpression of miR-17 could be observed in these cells for two weeks after transfection. Notably, the positive rate of CD8 was increased in these cells overexpressing miR-17 (Figure 4c). We next examined the existence of CD8+ cells in a grafted tumor microenvironment. In tumor-infiltrating T cells, we found higher percentages of CD8+ cells in the miR-17 transgenic mice compared with the wild type (Figure 4d).

### MiR-17 promoted proliferation of Jurkat cells co-cultured with B16 cells

**Figure 5:**
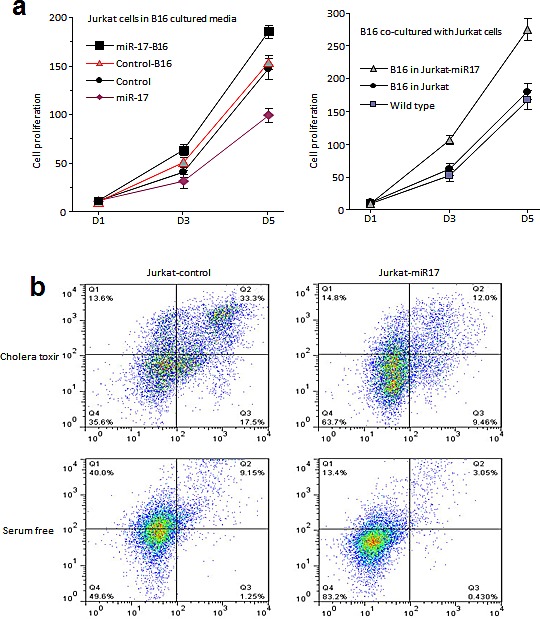
Co-culture of B16 and Jurkat cells (A) Jurkat cells overexpressing miR-17 grew significantly faster when co-cultured with B16 cells. (B) When miR-17 overexpressed Jurkat cells were treated with cholera toxin, they showed greater resistance to AICD and smaller number of cells underwent apoptosis. Jurkat cells overexpressing miR-17 survived better in serum-free media compared to the controls.

When Jurkat cells were co-cultured with B16 cells, they benefited each other in proliferation: Jurkat cells overexpressing miR-17 grew significantly faster when co-cultured with B16 cells (Figure 5a). These cells showed greater resistance to activation-induced cell death (AICD) as well (Figure 5b). When miR-17-transfected Jurkat cells were treated with cholera toxin, there were smaller number of cells undergoing apoptosis compared to controls (Figure 5b). Similarly, these cells survived better in serum-free media (Figure 5b).

**Figure 6:**
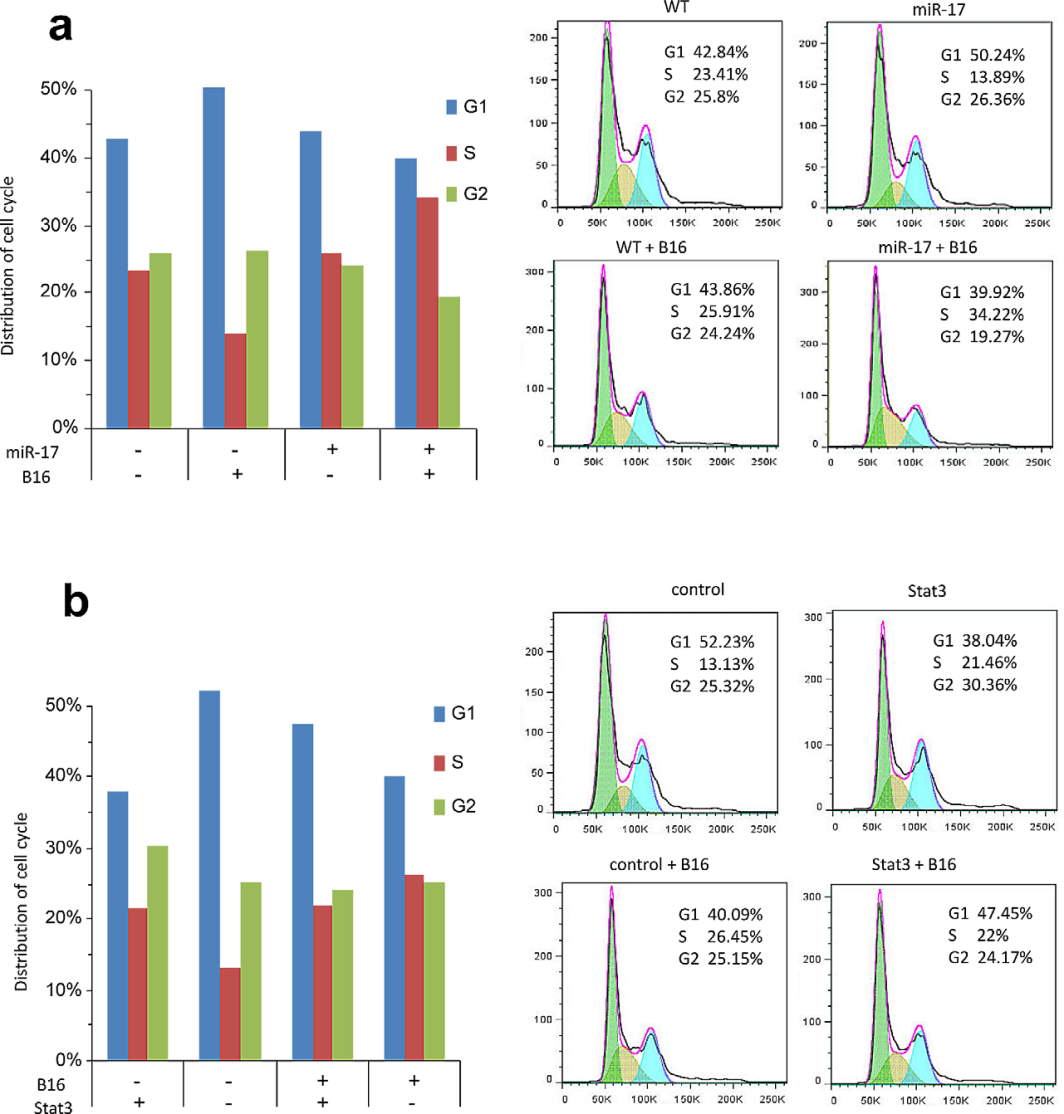
Cell cycles analysis in Jurkat cells with or without B16 co-cultured (A) Left, Without co-culture, miR-17 overexpression increased cell number in G1 phase compared with the control cells (50.24% vs. 42.84%). MiR-17 overexpressing cells in S phase also decreased (13.89% vs. 23.41%). When co-cultured with B16 cells, more cells overexpressing miR-17 were detected in S phase (34.22% vs.25.91%). (B) Silencing STAT3 reduced cell number in S phase (13.13%) but increased the number in G1 phase (52.23%), compared to 21.46% in S phase and 38.04% in G1 phase of cells transfected with a control oligo. When these cells were co-cultured with B16 cells, silencing STAT3 was associated with increased percentage of cells in S phase (26.45%) and decreased in G1 phase (40.09%).

Cell cycles were assayed in the miR-17-transfected Jurkat cells with or without B16 co-culture. When they grew independently, miR-17 overexpression in Jurkat cells increased the cell number in G1 phase (50.24%), compared to that in control group (42.84%) (Figure 6a). Consistently, miR-17 overexpressing cells in S phase also decreased to 13.89%, compared to 23.41% of control group (Figure 6a). However, when co-cultured with B16 cells, more cells overexpressing miR-17 were detected in S phase (34.22%). But there was only slightly increase in control cells (25.91%) (Figure 6a). To mimic the function of miR-17 *in vitro*, we further knocked down the expression of STAT3 by using siRNA against STAT3. As opposed to miR-17, knocking down STAT3 reduced the cell population in S phase (13.13%) and increased it in G1 phase (52.23%), compared to 21.46% in S phase and 38.04% in G1 phase of negative control oligos (Figure 6b). When these cells were co-cultured with B16 cells, suppression of STAT3 was associated with an increased percentage of cells in S phase (26.45%) and decreased in G1 phase (40.09%) (Figure 6b). There was no significant change observed in control cells. In summary, miR-17 targeted STAT3, promoted Jurkat-cell mitosis and proliferation during co-culture with melanoma B16 cells.

## DISCUSSION

The tumor microenvironment comprises blood vessels, immune cells, fibroblasts and the extracellular matrix. Numerous signaling molecules and pathways influence the interactions between the tumor and its surrounding microenvironment. It is believed that such interplay remodels the tumor microenvironment, permitting tumor angiogenesis and metastasis. Meanwhile, immune responses are often suppressed in the host, leading to tumor-tolerogenic macrophages, NK/T cells and neutrophils. Any fluctuation in the microenvironment could impact the global signaling of tumor cells, and thus influence the stress response through miRNA-regulated pathways. In our study, we found that microRNA-17 was able to target STAT3 in tumor microenvironment, thus inhibited melanoma tumor growth by stimulating the tumor infiltrating CD8+ T cells response.

There has been extensive research into the molecular mechanisms of tumor-mediated immune suppression, in an attempt to explain how tumor cells are able to escape the natural immune surveillance. It is becoming increasingly clear that the dysregulation of the immune response plays a critical role in cancer progression and therapeutic resistance. Hence, normalizing of the microenvironment can improve the body's ability to fight off cancer. Analysis of tumor infiltrating lymphocytes has demonstrated that many types of tumors show evidence of T-cell infiltration [10]. Of particular interest, activated CD8+ T cell responses have been associated with a positive prognosis in tumors such as colorectal cancer [11]. More studies are underway to explore the prognostic value of cancer-associated immune biomarkers. Recent findings have suggested that miRNAs are greatly involved in modulating the proliferation, differentiation and response of CD8+ T cells. Initial characterization of the miRNA profile in CD8+ T cells provided insight into the understanding of the role miRNAs play role in a cell-specific setting (Figure 4). Our previous study showed that CD8+ cells differentiation was impaired in miR-17 overexpression mice [7]. It could also be partially attributed to suppression of STAT3 [12]. In the absence of STAT3, T cells failed to mature into protective memory T cells [12]. Thus it is suggested that STAT3 drives a feedback loop to establish CD8+ T cells and other functional cell differentiation. In addition, many other signaling pathways are also actively involved in the regulation of T cell differentiation and clonal expansion, such as PTEN/PI3K/Akt and Wnt signaling [13, 14]. Since both pathways are under regulation of miR-17, the global immune suppression we observed in mice with miR-17 overexpression could be the result of a vastly complex mechanism of interconnected regulatory networks.

Interestingly, when melanoma cells were growing in the mice, effective immune response was observed in miR-17 overexpressing mice, leading to inhibited tumor development. Recent evidence has indicated microRNAs exert a fining tuning function to maintain cellular internal hemostasis [15]. MicroRNA-regulated stress response not only happens at the cellular level, but also mediates systemic reactions. In miR-17 transgenic mice, high levels of CD8+ T cells were detected in the spleen as well as peripheral blood. More importantly, they infiltrated into grafted tumors. It is generally recognized that CD8+ T cells play an important role in attacking tumor cells and impeding tumor growth. They directly mediate the death of tumor cells, and also produce inhibiting factors such as IFN-γ, TNF-α and IL-2. The combined effect is a driving force of anti-tumor immunity, especially in melanoma [16].

The understanding of the potent effects of miRNAs on tumor-mediated immunosuppression was driven by studies in tumor-bearing mice. However, the impact of microRNAs on anti-tumor immune response could be a double-edged sword. Increased expression of miR-15b was observed in isolated CD8+ T lymphocytes in mice with Lewis lung carcinoma [17]. Ectopic expression of miR-15b in CD8+ T cells inhibits apoptosis by knocking down death effector domain-containing DNA binding protein (DEDD). High expression of miR-15b is also associated with inactivation of CD8+ T lymphocytes by repressing the production of cytokines such as IL-2 and IFN-γ [17]. Despite its anti-apoptotic effect, miR-15b likely plays a negative role in the activation of effector T cells and anti-tumor immune response. Dynamic changes in tumor-associated miRNA expression have also been observed in the miR-17-92 cluster [18-20]. In patients with multiple myeloma, the miR-92a level in CD8+ T cells was significantly down-regulated compared with normal subjects [21]. With the remission of disease, the plasma miR-92a level became normalized. Given the fact that miR-92a and miR-17 belong to a same microRNA cluster, their roles in immune mediation could be alike. It is notable that miR-17-92′s function in tumor growth and progression still remain controversial, which mainly display in a cell-specific context. Their levels are generally elevated in leukemia but suppressed in breast cancer [22]. In contrast, both miR-17 and miR-92a promote immune cell mediated anti-tumor response. It is therefore suggested their regulations of tumor development and progression are multilayered and through different mechanisms.

Accumulating evidence has identified STAT3 as a critical molecule in regulating tumor-associated immunosuppression by interfering with multiple factors. Constitutive expression of STAT3 alters gene-expression programs, inhibits expression of immune mediators and suppresses leukocyte infiltration into the tumor [23]. Blocking STAT3 in immune cells can generate diverse anti-tumor immunity by suppressing negative regulators such as immature dendritic cells and regulatory T cells and activating CD8+ T cells, natural killer cells and neutrophils [23]. Thus, STAT3 has emerged as a potential target for tumor immunotherapy. Recent studies have demonstrated that the interplay between miRNAs and STAT3 broadly exists in cancer development and progression. MiR-124 has been reported as a potential tumor suppressor in diverse tumor types, such as colorectal cancer and prostate cancer [24]. In patients with glioblastoma, the expression of miR-124 is significantly reduced, compared to normal brain tissues [25]. Ectopic up-regulation of miR-124 in glioma stem-like cells promoted T cell proliferation and regulatory T cell induction. Moreover, treatment of T cells from glioblastoma patients with miR-124 induced pro-inflammatory cytokines and chemokines [25]. As a result, systemic administration of miR-124 prolonged overall survival and decreased tumor incidence in a murine glioma model. Such anti-tumor effects were shown to be dependent on the presence of T cells. In tumor bearing mice depleted of CD4+ or CD8+ cells, the immunotherapeutic effects of miR-124 was ablated [25]. Jurkat cell is a well-established model to investigate microRNA function. Our findings demonstrated that forced expression of miR-17 in Jurkat cells promoted cell proliferation and survival in the presence of B16 cells. Moreover, inhibition of STAT3 expression can achieve the same effect as miR-17 over-expression. The STAT3 pathway has been extensively studied in Jurkat cells, and these cells have the potential to differentiate into subtypes of T cells [26]. Upon differentiation, there was a significant down-regulation in the expression of STAT3 [27]. Thus it is suggested that miR-17 promotes Jurkat cell differentiation *in vitro*, by targeting STAT3.

Activation of STAT3, in turn, can modulate expression of several miRNAs. For example, there is a highly conserved STAT3-binding site in the promoter of the miR-17 (C13orf25) [28]. By modulating the expression of IL-6, activation of STAT3 upregulates the entire miR-17-92 cluster. Our finding also confirmed that the 3′-UTR of STAT3 harbors a miR-17 binding site and is subject to negative regulation of miR-17. By modulating STAT3 associated immune response in tumor microenvironment, the negative regulatory loop between miR-17 and STAT3 may be an important factor in tumor-associated immune tolerance and a potential immunotherapeutic target against cancer.

## MATERIAL AND METHODS

### Cell culture

Jurkat cells (TIB-152) were cultured in RPMI-1640 medium with 10% fetal bovine serum (FBS). B16 cells (CRL-6475) were cultured in DMEM medium with 10% FBS. Cells were maintained at 37°C with 5% of carbon dioxide. Fresh medium was added/changed every 2 to 3 days.

### Generation of transgenic mice

The transgenic mice were developed by the microinjection of a miR-17 overexpression plasmid into C57BL/6 mice zygotes [7]. Then the fertilized embryo was implanted into a female recipient's uterus. F1 mice were backcrossed with wild type C57BL/6 mice and positive offspring were identified by genotyping PCR. All animal experiment protocols were approved by the Animal Care Committee of Sunnybrook Research Institute, Ontario, Canada.

### Flow cytometry

Peripheral blood cells were obtained by heart puncture and spleen cells were isolated by using a cell strainer (Fisherbrand). Live cells were suspended in phosphate buffered saline (PBS) and counted using a hemocytometer (Bright-Line). Cells were incubated with FITC-conjugated anti-mouse CD4 (Caltag Laboratories), PE-conjugated anti-mouse CD8 (BD Biosciences) and PerCP-conjugated anti-mouse CD45 (BD Biosciences) for 30 minutes before resuspension for analysis. FACScan flow cytometer (BD Biosciences) were used and the data were analyzed using FlowJo software.

In the cell apoptosis assay, 1 × 106 cells were washed twice in PBS before re-suspension in 50 μL of HBSS with 2% calf serum. 5 μL of Annexin V-FITC and Propidium Iodide (BD Pharmingen) was added and then placed on ice for 30 minutes. The cells were re-suspended to 500 μL of HBSS, followed by flow cytometry analysis within 30 minutes.

In cell cycle analysis, co-cultured cells were harvested and washed twice with PBS. Cell number was adjusted to 2 × 106/mL in 50 μL of HBSS with 2% calf serum. The cells were then incubated with 1 mL of 80% ice cold ethanol for 30 minutes. Propidium Iodide (Sigma) and 0.6% of NP-40 were added into the cell suspension, followed by DNA content analysis by flow cytometry as described [29].

### Immunohistochemistry

Tumor xenograft and spleen were harvested from mice after B16 cell intraperitoneal injection. All of the antibodies were purchased from Abcam. Mouse antibodies against CD4, CD8 and STAT3 were employed as primary antibodies and biotinylated goat anti mouse IgG was used as secondary antibody. The assay was performed as described [30]. Each slide was assigned a score for density and intensity.

### Western blotting

Cultured cells or animal tissue lysates were prepared for SDS-PAGE electrophoresis. Western blotting was subsequently performed as previously described [31].

### Luciferase assay

Luciferase activity assays were performed as previously described [32, 33]. In brief, U343 cells were seeded onto 12-well tissue culture dishes at a density of 1 × 10^5^ cells/well and co-transfected with the luciferase reporter constructs and miR-17-5p mimic with Lipofectamine 3000 (Life Technologies). After overnight incubation, the cell lysate was prepared with buffer from Dual-Luciferase® Reporter Assay Kit (Promega). Luciferase activity was detected by a microplate luminescence counter (Perkin Elmer).

### Cell proliferation assay

B16 cells were co-cultured with Jurkat cells transfected with GFP mock control or miR-17 overexpression plasmid. Cells were plated at a density of 1 × 105 cells/well in DMEM containing 10% FBS and maintained for 5 days. The cells were harvested and cell number was counted at different time points. The assay was performed as described [34].

### Statistical analysis

All experiments were performed at least three times. Numerical data were subject to independent sample *t* test. Categorical data were subject to Pearson's chi-squared test. The statistical significance was set at *p<0.05 and **p<0.01.
